# Static Tactile Sensing for a Robotic Electronic Skin via an Electromechanical Impedance-Based Approach

**DOI:** 10.3390/s20102830

**Published:** 2020-05-16

**Authors:** Cheng Liu, Yitao Zhuang, Amir Nasrollahi, Lingling Lu, Mohammad Faisal Haider, Fu-Kuo Chang

**Affiliations:** 1 Department of Mechanical Engineering, Stanford University, Stanford, CA 94305, USA; 2 Zenith Aerospace, Redwood City, CA 94063, USA; eblistx@gmail.com; 3 Department of Aeronautics and Astronautics, Stanford University, Stanford, CA 94305, USA; amirnasr@stanford.edu (A.N.); fhaider@stanford.edu (M.F.H.); fkchang@stanford.edu (F.-K.C.); 4 Key Laboratory for Mechanics in Fluid Solid Coupling Systems, Institute of Mechanics, Chinese Academy of Science, Beijing 100190, China; lulingling@imech.ac.cn

**Keywords:** robotic tactile sensing, electronic skin, piezoelectric sensors, static pressure load sensing, electromechanical impedance-based method

## Abstract

Tactile sensing is paramount for robots operating in human-centered environments to help in understanding interaction with objects. To enable robots to have sophisticated tactile sensing capability, researchers have developed different kinds of electronic skins for robotic hands and arms in order to realize the ‘sense of touch’. Recently, Stanford Structures and Composites Laboratory developed a robotic electronic skin based on a network of multi-modal micro-sensors. This skin was able to identify temperature profiles and detect arm strikes through embedded sensors. However, sensing for the static pressure load is yet to be investigated. In this work, an electromechanical impedance-based method is proposed to investigate the response of piezoelectric sensors under static normal pressure loads. The smart skin sample was firstly fabricated by embedding a piezoelectric sensor into the soft silicone. Then, a series of static pressure tests to the skin were conducted. Test results showed that the first peak of the real part impedance signal was sensitive to static pressure load, and by using the proposed diagnostic method, this test setup could detect a resolution of 0.5 N force. Numerical simulation methods were then performed to validate the experimental results. The results of the numerical simulation prove the validity of the experiments, as well as the robustness of the proposed method in detecting static pressure loads using the smart skin.

## 1. Introduction

Tactile sensing in human and animal skins enables them to touch, sense temperature, etc. The haptic perception, if added to robots, can significantly enhance their performance through better human-robot and robot-environment interactions. In comparison, even the most sophisticated robots have at most a few dozen tactile sensors. Regardless of over thirty years of research, tactile sensing still falls behind progress in computer vision methods. The reason for this discrepancy is that compared to cameras, tactile sensors must be compliant, tough and flexible enough to coat the surfaces of robotic limbs and hands. In addition, as the number of sensors increases, wiring and signal transfer become a major issue [1,2,3,4,5].

**Figure 1:**
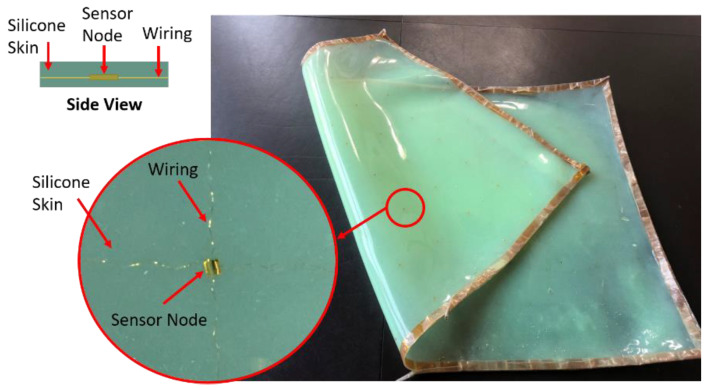
Robotic electronic skin (a.k.a. smart skin) with embedded multi-modal sensor network.

To overcome the aforementioned challenges for robotic tactile sensing, Stanford Structures and Composites Laboratory (SACL) developed a smart skin shown in Figure 1 by embedding a multi-modal stretchable sensor network [6] into a soft silicone. Guo [7] has documented in detail the fabrication and material selection process of this skin. This artificial skin has been added to a robotic arm for realizing autonomous control, which leverages advanced sampling-based motion planning techniques [7]. Utilizing the signals of the multi-modal sensors, which are embedded in the skin in state awareness algorithms as input parameters, the robotic arm can sense and react to environmental changes such as temperature variance and local dynamic impacts onto the skin [7]. However, one critical aspect in tactile sensing has not been well studied, which is how to detect static pressure in the smart skin using lead zirconate titanate (PZT) elements, also known as piezoelectric sensors.

There exist different tactile sensors for measuring static pressure and contact conditions using different transduction mechanisms [8,9,10,11]. Resistive-based tactile sensors can reach high sensitivity, but have high power consumption and lack the measurement of contact forces [12]. Optical tactile sensors are also capable of reaching high sensitivity, although they will show loss of light due to micro bending chirping. Meanwhile, power consumption is also a big challenge [12]. Triboelectric tactile sensors have the advantage of being self-powering, although their long-term unreliability is still an issue [13]. One of the most common tactile sensors are capacitive tactile sensors, which can achieve high sensitivity [4]. However, noises coming from temperature and humidity variations, and even electrical noise introduced by unshielded power supplies, may significantly decrease the capacitive signal-to-noise ratio. This issue imposes a signal conditioning stage in order to obtain a high signal-to-noise ratio, which results in complex circuitry [4]. In addition, under highly repetitive loads, the capacitive sensors are prone to failure due to mechanical fatigue, which undermines the reliability of the method [14].

Compared with capacitive sensors, PZT sensors are excellent in terms of mechanical robustness and their simplicity of use and noise resistance. Piezoelectricity in PZT is a well-known transduction mechanism for dynamic force measurement by using the direct piezoelectric effect. However, a different mechanism is required to sense static loads using PZT sensors. One promising method is to use PZT sensors in a resonant piezoelectric sensing mode. Safour and Bernard [15] demonstrated that the measured electric admittance spectrum of a PZT sensor can be correlated to the static forces applied to the PZT sensor. Although their study was focused on the applied force in the order of 100 N, which is relatively high, and applied directly on the PZT sensor, their results showed the possibility of this method for tactile sensing. In general, tactile sensing requires a higher resolution, and sensors must be embedded in a skin material. Such inclusion of the sensors inside skin material like silicone rubber or fiberglass can prevent sensors from direct contact with external environments, which may damage the sensors. Shin [14] also investigated the effect of static load on PZT sensors by using impedance measurement. Their results showed a decrease in the impedance value at anti-resonance frequencies, with increasing applied load. However, their work demonstrated a force resolution of 10 N, and has not been proven theoretically or numerically. Ozeri [16] also experimentally investigated the static force measurement by PZT sensors. However, the minimum detectable load in their work was still very large, at about 17.67 N (25 kPa on a circular area of 30 mm in diameter). In this paper, an electromechanical impedance (EMI)-based method is used as the diagnostic approach to investigate the ability of the proposed smart skin for sensing static pressure loads. Differently than other studies, however, we especially focus on the detection for small amounts of normal loads (0.5 N as the resolution). Furthermore, such active sensing methods are considered to be more simplified than passive sensing, as they eliminate the required power supply throughout the operation time.

## 2. Problem Statement

As the shortcomings of the current sensing approaches in robotic smart skin application impede high-resolution and robust tactile sensing, in this study an electromechanical impedance (EMI)-based approach is proposed to be implemented. The hypothesis is that the amplitude of the impedance response of lead zirconate titanate (PZT) sensors, as the sensing elements, depends on the contact properties of the skin. Considering the PZT sensor’s mechanical robustness and simplicity to use, the objective of this work is to study the capability of the PZT sensor in the smart skin to detect static pressure loads, which is a critical portion of robotic tactile sensing. The EMI-based approach is used by correlating the impedance signal directly to static pressure load. The resolution and sensitivity of this method are then evaluated. A special effort is emphasized on the numerical simulation investigation to physically validate the relationship between the impedance signal variation and the applied static pressure load.

## 3. Method of Approach

**Figure 2:**
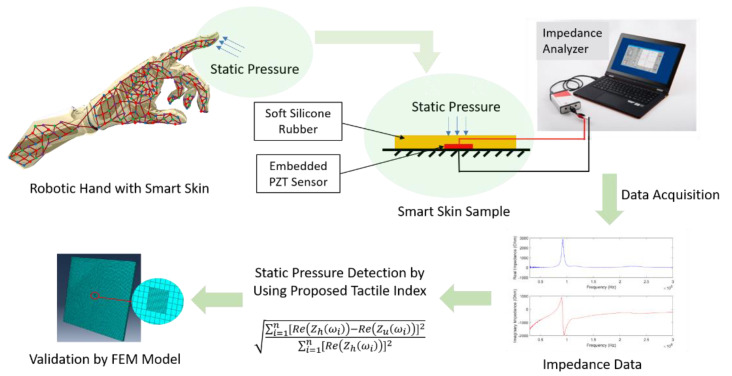
The framework of using the EMI-based method to detect static pressure loads for application on smart skin with an embedded PZT sensor.

The goal of this work is to study the feasibility of detecting static pressure loads using lead zirconate titanate (PZT) sensors embedded in the smart skin by an electromechanical impedance (EMI)-based approach. In order to achieve this objective, as shown in Figure 2, the method of approach can be divided into the following tasks: (1) developing the smart skin sample with sensors and silicone rubber, emulating the skin material; (2) measuring the electromechanical impedance of the sensors under various static pressure loads applied to the sample; (3) signal processing to correlate the impedance response to the applied static pressure loads; and (4) validating the resonance behavior of the sensor embedded in the skin by numerical and analytical methods.

## 4. Experiment and Results

### 4.1. Smart Skin Sample Development

**Figure 3:**
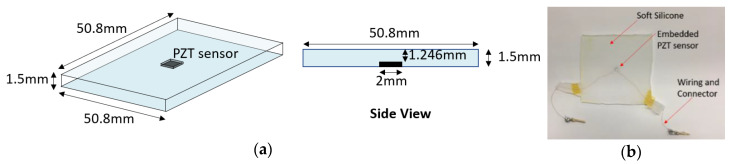
(**a**) Schematic of smart skin sample with a PZT sensor embedded in soft silicone; (**b**) the real sample with wiring and connectors.

The development of the smart skin sample is presented in this section. The 3D schematic of a smart skin sample with an embedded lead zirconate titanate (PZT) sensor is shown in Figure 3a. This sensor, whose dimensions were 2 mm × 2 mm × 0.254 mm, was fabricated by APC ceramics. The material properties of this PZT sensor can be found in Table 1. The wired sensor was then embedded in the skin-like soft silicone rubber material (Smooth-On Ecoflex 00-30 [17]), as illustrated in Figure 3b, to form a smart skin sample. This sample was 50.8 mm by 50.8 mm, with a thickness of 1.5 mm.

### 4.2. Test Procedure

**Figure 4:**
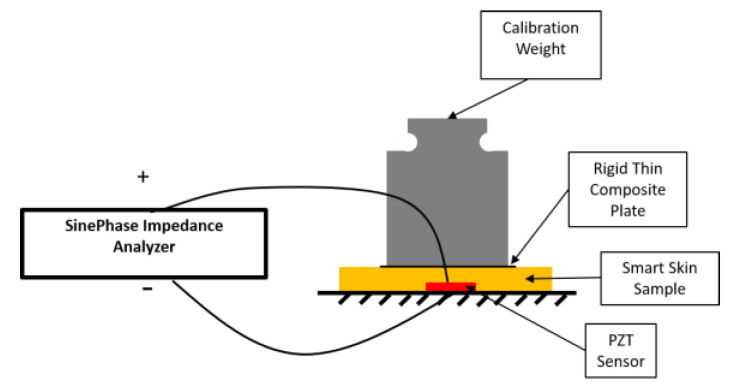
Schematic of the experimental test setup.

**Figure 5:**
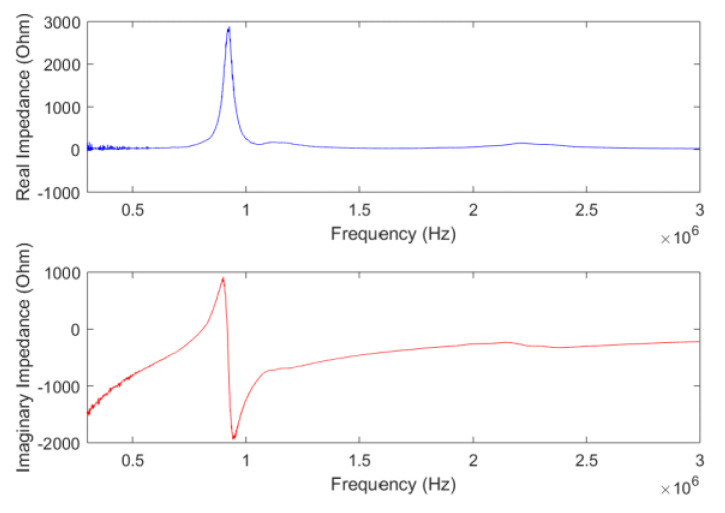
The electromechanical impedance behavior from the PZT sensor at zero static pressure load.

To apply the static pressure load, in each instance a 50 g calibration weight was gently placed on top of the embedded PZT sensor, as shown in Figure 4. To guarantee a uniform pressure, a rigid thin composite plate with dimensions of 20 mm by 20 mm was placed between the skin and the calibration weight. Two wires (AWG 40) of 80 µm in diameter embedded in the silicone skin extended the top and bottom electrodes of the PZT sensor, respectively, to the outside of the skin, which were then connected to a PC-connected impedance analyzer (SinePhase Impedance Analyzer 16,777 k). This impedance analyzer then actuates the PZT sensor over a designated range of frequencies, and collects the impedance response accordingly over this frequency range. In the experiment, the impedance signal was recorded at each load increment. One of the collected impedance signals for 100 kHz to 3 MHz, with an increment of 1 kHz, is shown in Figure 5. Both the real and imaginary parts of the recorded data are shown in Figure 5; however, only the real part was used for later analysis. The first resonant peak is around 900 kHz, which corresponds to the radial vibration modes of the PZT sensor.

### 4.3. Results of Impedance Response to Static Pressure Loads

**Figure 6:**
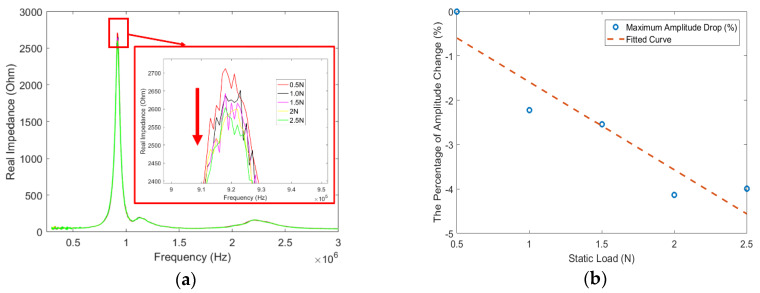
(**a**) Real part of the impedance response under different static pressure load; (**b**) maximum impedance amplitude change at each static load with respect to the value at 0.5 N.

In this study, we focused on the real part of the impedance response. As illustrated in Figure 6a, in general, a gradual decrease in the amplitude at the first peak frequency happens with the increment of the static pressure load. The maximum amplitude value at each load was extracted. The percentage change of these values at each load, with respect to the maximum amplitude value at 0.5 N, were plotted in Figure 6b. Directly comparing the maximum amplitude under each load is the traditional method that has been previously investigated and applied at large load levels [14,15]. In our case, however, since the load level is as small as 0.5 N, this direct comparison of maximum amplitude does not work well. It can be observed that although there is a decreasing trend, there exists inconsistency at the load of 2 N. This is due to the mismatch between this high-resolution load and the limited sampling rates of the data acquisition system.

To overcome this challenge, a novel diagnostic method was proposed by using the root mean square deviation (RMSD) [18] of Equation (1) as the tactile index (TI) to evaluate the static pressure load:

(1) $$TI=\sqrt{\frac{\sum_{i=1}^{n}{\left[Re(Z_{h}(\omega_{i}))-Re(Z_{u}(\omega_{i}))\right]}^{2}}{\sum_{i=1}^{n}{\left[Re(Z_{h}(\omega_{i}))\right]}^{2}}},$$

In this equation, 𝑍_ℎ_(𝜔_𝑖_) denotes the baseline impedance; 𝑍_u_(𝜔_𝑖_) is added weight impedance; and 𝜔_𝑖_ denotes frequency interval. 

**Figure 7:**
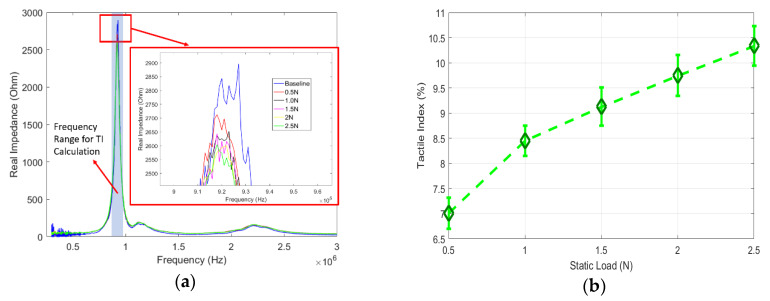
(**a**) Real part of the impedance response under different static pressure loads, including baseline data; (**b**) tactile index at different static loads.

To apply this TI, baseline data were selected as the impedance response at 0 static loads, as shown in Figure 7a. Note that there is a perceivable difference between the baseline signal and the signals under static loads. This is due to the different force boundary conditions in these two different scenarios. This tactile index is essentially describing the average impedance change from the baseline signal at each corresponding load. By using this definition, the applied frequency range is centered in the first peak frequency within a range of 50 kHz, as shown in Figure 7a. Note that to collect data over this 50 kHz frequency range, the response time was about 600 µs. The TI values were then calculated under each static load and plotted in Figure 7b with error bars, which was based on five individual tests. It is observed that with the increment of static pressure load, the TI values consistently increase, and are highly distinguishable for each force increment of 0.5 N, which was the minimal detectable force in this current setup, with a sensitivity of 1.8%/N. This resolution is comparable to capacitive sensors, and proves its great sensitivity in terms of detecting the static pressure load using the proposed diagnostic method.

## 5. Finite Element Simulation Study

**Figure 8:**
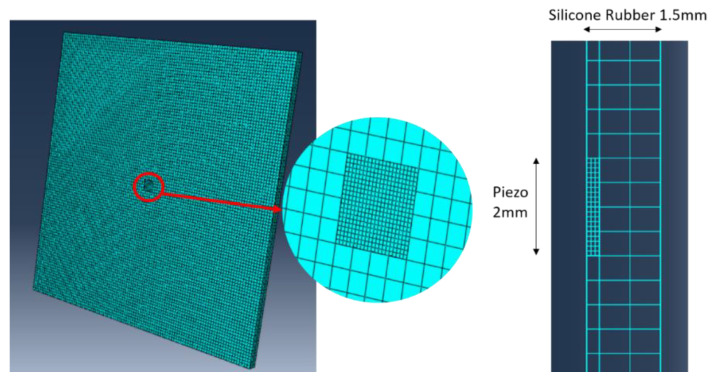
The FEM model and mesh of the smart skin sample with an embedded PZT sensor.

To model the electromechanical impedance (EMI) response of the embedded lead zirconate titanate (PZT) sensors, a finite element model (FEM) was created using the commercial software Abaqus 6.12. The objective of the simulation was to obtain qualitative insights into the resonant behaviors of the embedded PZT sensors under the applied static pressure loads. The dimensions of the model were identical to what is shown in Figure 3, and the mechanical material properties of this piezoelectric material are shown in Table 2. The direct piezoelectric matrix [d] and permittivity [e] are shown in matrix (2) and (3) respectively. The C3D8E elements, which stand for solid elements with built-in piezoelectric properties, were applied to model the PZT sensor. The mesh size of the PZT sensor was set to 100 µm, based on the previous convergence study [18]. The EMI of the PZT sensor was calculated over the frequency domain from 0.5 MHz to 1.5 MHz, with an interval of 10 kHz. Considering this frequency range and the typical mechanical wave speed in silicone rubber, which is around 1000 m/s [19], the wavelength of acoustic waves in the silicone rubber material varies from 0.67 mm to 2 mm. Therefore, the mesh size of the silicone was chosen as 0.5 mm to ensure that it was fine enough to capture the response over the desired frequencies. The FEM model and mesh sizes are shown in Figure 8. The interaction between the PZT sensor and silicone was defined in both tangential and normal direction: the tangential behavior was defined with a friction coefficient of 0.9 [20], and the normal behavior was set as “Hard” contact.

(2) $$d=\left[\begin{array}{cccccc} 0 & 0 & 0 & 0 & 584 & 0\\ 0 & 0 & 0 & 584 & 0 & 0\\ -171 & -171 & 374 & 0 & 0 & 0 \end{array}\right]\times 10^{-12}CN^{-1},$$

(3) $$\varepsilon_{\sigma }=\left[\begin{array}{ccc} 1730 & 0 & 0\\ 0 & 1730 & 0\\ 0 & 0 & 1700 \end{array}\right]\times \varepsilon_{0},$$

### 5.1. Hyperelastic Material Property of Silicone Rubber

**Figure 9:**
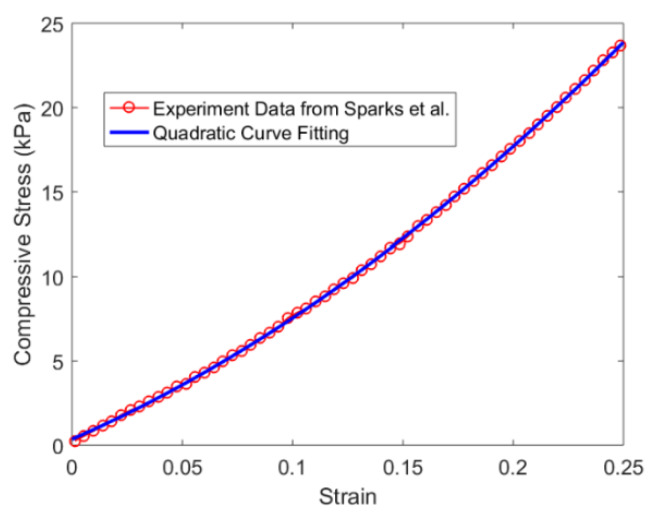
Nonlinear stress-strain relationship for Smooth-On Ecoflex 00-30 under static compressive load.

One key factor dominating the impedance behavior of the embedded PZT sensor is the hyperelastic material property of the silicone rubber material. The stress-strain relationship of hyperelastic materials such as silicone rubber is normally defined as nonlinear elastic and incompressible [17,21]. Sparks et al. [17] demonstrated the stress and strain relationship shown in Figure 9 under a static compressive load for the Smooth-On Ecoflex 00-30, which is the silicone rubber used in our experiment. It shows a clear nonlinear behavior, which indicates that the stiffness of this silicone material increases with the applied load.

Therefore, to correlate the stress level to the silicone’s stiffness change, the stress and strain relationship from Sparks’ experiment result was fitted into a quadratic curve, which is also plotted in Figure 9. This quadratic curve is presented explicitly in Equation (4), where S denotes stress and ε denotes strain. The stresses at each load increment in the experiment were used to calculate the corresponding strains using Equation (4). These strain values were then used to obtain the corresponding stiffness as the slope of the quadratic curve at each stress level. Table 3 shows the values of stress, strain and stiffness at each load increment. Note that the weight of the rigid composite plate, which is 1.5 g and used for even pressure distribution, was also taken into consideration.

(4) $$S=147.7\varepsilon^{2}+57.14\varepsilon +0.3583\;kPa,$$

### 5.2. Dynamic Compressive Behavior of Silicone Rubber Material

The stress-strain relationship in Figure 9 was measured under a static compressive load. In our simulation, however, the interaction between the PZT sensor and silicone rubber was highly dynamic at the frequency level of megahertz. Therefore, the silicone rubber was driven by the PZT sensor under dynamic strains. This means that this stress-strain curve for the silicone rubber needs to be modified, considering this load rate effect. In highly viscous materials such as silicone rubber, the stress-strain curve is greatly dependent on the applied strain rates. Song et al. [22] experimentally determined the dynamic compressive stress-strain relationships of an ethylene propylene-diene monomer copolymer (EPDM) rubber at various strain rates. Their test results showed that at the true strain of 0.1, as the strain rate increases from 653 Hz to 4730 Hz, the true stress increases from 1.0 MPa to 7.0 MPa, which is seven times higher in stiffness. It is also observed that the nonlinear behavior of the hyperelastic materials still holds, regardless of the increase of the strain rate. Based on the fact from this study that with the 7.24 times increase of the strain rate, the stiffness increased by seven times. Therefore, in our simulation, the stiffness values in Table 3 were amplified by the same amount. This amplification factor is already quite conservative, considering that the frequency range in our simulation is on the orders of 10^6^ Hz. Again, the objective of this simulation study was to obtain qualitative insights into the impedance behavior of the embedded PZT sensor. Therefore, by amplifying seven times, the stiffness was good enough to capture the change of the impedance behavior.

Taking into account both the hyperelastic property and dynamic effect on the silicone rubber stiffness, in order to simulate the static pressure load effect onto the smart skin sample, the following stiffness values were used to represent each corresponding loading status, as shown in Table 4.

### 5.3. A Direct Steady-State Dynamic Analysis for Simulating Impedance Behavior

**Figure 10:**
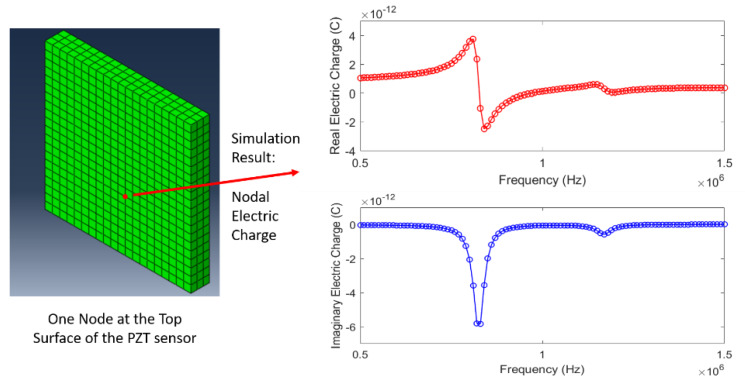
Simulation result of the nodal electric charge at one node at the top surface of the PZT sensor.

A direct steady-state dynamic analysis was performed using Abaqus 6.12. A zero displacement in the *x*, *y* and *z* directions on the bottom surface of the skin was considered as mechanical boundary conditions. The following electrical boundary conditions were applied to the sensor: a constant voltage of 1 V in magnitude and 0 in phase at the top surface electrode of the PZT sensor, while the bottom surface electrode was fixed as the ground of 0 V. For each simulation, the nodal electric charges at the top surface electrode were extracted and summed to compute the total electrical charge (Q). Figure 10 shows the simulation result of the nodal electric charge at one node at the top surface. Again, the total electric charge (Q) is the summation of these nodal electric charges over all the nodes at the top surface of the PZT sensor.

After obtaining the total electric charge (Q), the current in the transducer can be determined by the following equation

(5) I=iωQ,

where ω is the angular frequency of the applied actuation voltage.

The impedance was then calculated using Equation (6)

(6) $$Z=\frac{V}{I},$$

where V is the voltage difference across the PZT sensor, which was 1 V. Note that the impedance is in a complex form.

**Figure 11:**
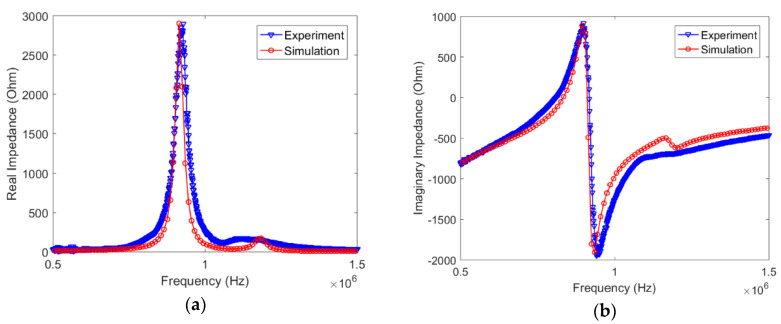
Comparison of the experiment and simulation results on the impedance behavior of a PZT sensor embedded in the silicone rubber: (**a**) real part; (**b**) imaginary part.

Figure 11 illustrates the simulation results, including both real and imaginary parts of the impedance response at baseline state (0 pressure load). For comparison, the experimental results were also plotted in the figure, and show good agreement with the simulation results. Here, in order to match the amplitude of the first resonant peak in the experiment, a structural damping coefficient of 0.04 for the PZT sensor was used in the simulation. Although the damping coefficient is very difficult to be accurately determined by experiments [18], its value used in this simulation was assumed to be the closest estimation towards our experiment. Again, the purpose of this numerical study was to gain a qualitative understanding about the effect of normal pressure loads to the impedance behavior of the PZT sensors embedded in the silicone rubber.

### 5.4. The Effect of the Silicone Rubber Stiffness to the Impedance Response

**Figure 12:**
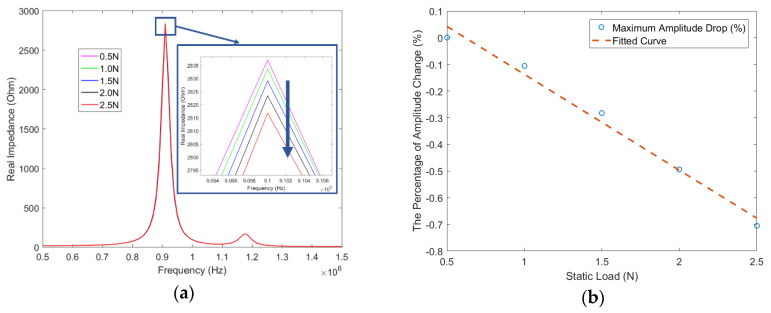
(**a**) Simulation results of the static normal pressure load effect to the smart skin sample with a PZT sensor embedded in the silicone rubber; (**b**) simulated maximum amplitude change at each static load, with respect to the value at 0.5 N.

To study the effects of changing the stiffness of the silicone rubber, different dynamic stiffness values at each static normal pressure level were used in the numerical model, and the corresponding impedance response was then obtained for each stiffness state. Figure 12a shows the real part of the impedance responses at each normal pressure state. The impedance peak decreases with the increase of the static normal pressure. As shown from Figure 12b, a similar trend was observed in the experiment results in Figure 6b. This verified our assumption that the static normal pressure load changed the stiffness of the hyperelastic silicone material, which eventually contributes to the impedance behavior change of the embedded PZT sensor.

## 6. Discussions Based on the Theoretical Model

### 6.1. Analytical Model: Dynamic Interaction between the PZT Sensor and Structure

**Figure 13:**
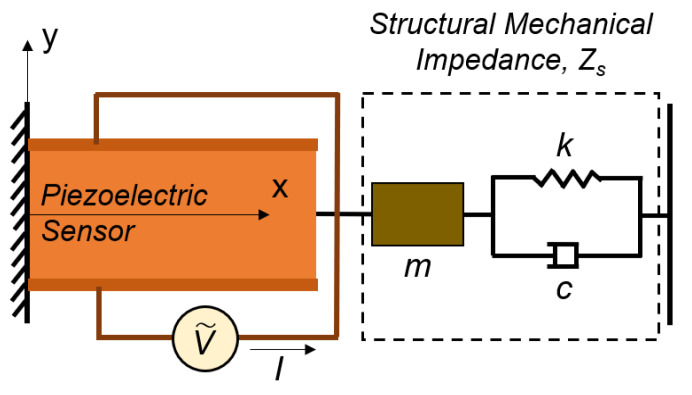
Electro-mechanical coupling between the PZT sensor and the structures.

Liang et al. [23] developed a 1D model to describe the interaction between a lead zirconate titanate (PZT) sensor and a structure. The essence of this impedance-based model is illustrated in Figure 13. Their results show that the complex electromechanical admittance Y, which is the inverse of electromechanical impedance, can be represented by Equation (7), in which ω is the angular frequency; w, l and h are the width, length and thickness of the PZT sensor, respectively; d_31_ is the piezoelectric strain coefficient; ε_33_ is the dielectric permittivity; and Y^E^ is the Young’s Modulus of the PZT sensor. The structural mechanical impedance Z_S_ is shown in Equation (8), where c is the damping coefficient and the mechanical impedance of the PZT sensor Za is represented in Equation (9)

(7) $$Y=2\omega i\frac{wl}{h}[\varepsilon_{33}-d_{31}^{2}Y^{E}+(\frac{Z_{a}}{Z_{s}+Z_{a}})\;\;d_{31}^{2}Y^{E}(\frac{\tan\;kl}{kl})],$$

(8) $$Z_{s}=c+\omega im(1-\frac{\omega_{R}^{2}}{\omega^{2}}),$$

(9) $$Z_{a}=\frac{kwhY^{E}}{\omega i\tan kl},$$

where $\omega_{R}=\sqrt{\frac{k}{m}}$ and $k=\omega \sqrt{\frac{\rho }{Y^{E}}}$.

### 6.2. The Effect of Stress on PZT Material Properties to Impedance Response

Since in Equation (7), Y is a function of the PZT properties, it is necessary to investigate the stress effects to these parameters to evaluate its influence on the impedance response. Zhang et al. [24] studied the effect of mechanical stresses on the responses of PZT sensors. Both the soft-type PZT and hard-type PZT were selected in their study. Their results show that for the hard PZT ceramics under compressive uniaxial stress T_3_, with the increase of T_3_ from 0 MPa to 75 MPa, the piezoelectric strain coefficient d_31_ increased proportionally by around 50%, and the dielectric permittivity ε_33_ also increased almost linearly by 60%. However, considering that the compressive stress level in our study is in the order of 10^−2^ MPa, the stress effect to the coefficient of d_31_ and ε_33_ is negligible. In addition, in both studies of Zhang et al. [24] and Safour et al. [15], the Young’s Modulus Y^E^ has even less change under the uniaxial compressive stress compared with d_31_ and ε_33_, so that its change can also be ignorable. Therefore, in this study, the effect of stress on PZT material properties to impedance response is insignificant.

### 6.3. The Effect of Stress on PZT Geometry Change to Impedance Response

**Figure 14:**
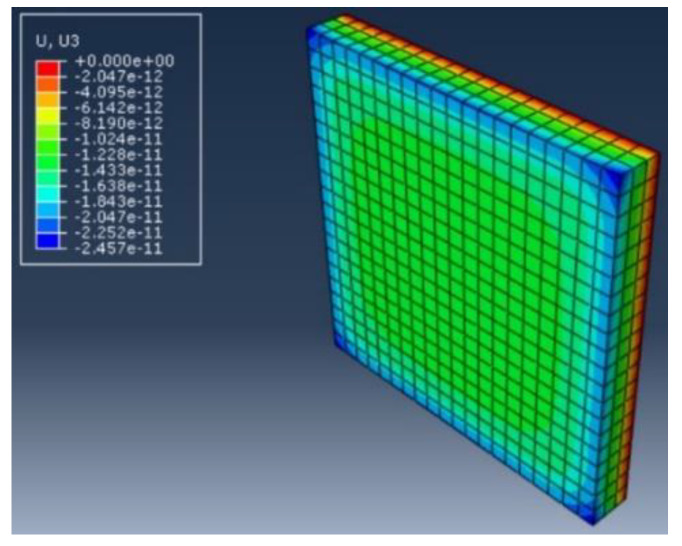
Displacement distribution of a PZT sensor in thickness direction under a static pressure load of 6.29 kPa. (U and U3 are displacement in m.).

Since in Equation (7), Y is also a function of PZT geometry, it is necessary to investigate the stress effects to variations of PZT geometry to evaluate its influence on the impedance response. More specifically, geometry parameters of w, l and h—which are the width, length and thickness of the PZT sensor—were taken into consideration. Finite element model (FEM) simulation analysis of the PZT sensor under static normal pressure was performed. The material properties, element type and mesh size were the same as described in the impedance simulation section. The bottom surface of the PZT sensor was applied a mechanical boundary condition with fixed displacement in all three directions. The top surface of the PZT sensor was applied a static normal pressure load of 6.29 kPa, which is the maximum load shown in Table 3. Figure 14 shows the displacement distribution in the thickness direction. The maximum displacement in the thickness direction is −2.457 × 10^−11^ m. For the width and length direction, the maximum displacements are 1.621 × 10^−11^ m and 1.885 × 10^−11^ m, respectively. By plugging these changes into Equation (7), we found that the overall changes to the impedance response are at the level of 0.0002%. This change is essentially extremely small compared with the experiment results, which have a change of 5% in the amplitude decrease. Therefore, in this study, the effect of stress on PZT geometry changes to impedance response can be neglected.

## 7. Conclusions and Future Work

Based on the robotic electronic skin technology developed by Stanford Structures and Composites Laboratory, this work is focused on the study of tactile sensing for static pressure using an electromechanical impedance-based method. A smart skin sample with an embedded lead zirconate titanate (PZT) sensor was fabricated, and a set of static pressure load experiments were performed. The collected impedance data from the embedded PZT sensor showed a consistent decrease in the amplitude of first peak in the real part impedance, with the increase of the applied static pressure load. A diagnostic method was proposed, which can successfully distinguish a force resolution of 0.5 N. A numerical simulation was performed to verify the change of the impedance response in the experiment, which is closely related to the stiffness change of the silicone rubber due to load effects. Finally, based on the theoretical model, different effects for the impedance response were discussed. Again, the stiffness change of the silicone rubber skin due to applied static pressure was proven to be the dominant factor for the impedance change in this study.

Future work includes: (a) applying this transduction mechanism to a real robotic gripper to realize the dexterous manipulation of pick and drop; (b) investigating higher static pressure load range, where not only the silicone material property will change, but so too will the piezoelectric material property; and (c) studying the impedance response to other load conditions, such as non-uniform stress.

## Figures and Tables

**Table 1:** The properties of the piezoelectric material in APC plate sensor.

Density	Young’s Modulus E_11_	Young’s Modulus E_33_	Relative Dielectric Constant K_T_	Piezo Charge Constant d_33_	Piezo Voltage Constant g_33_
7.6 g/cm^3^	63 GPa	54 GPa	1700	400 pC/N	24.8 mV-m/N

**Table 2:** The mechanical material properties used in the numerical simulation.

Property	Unit	Piezo PZT-5A
E_11_	GPa	60.97
E_22_	GPa	60.97
E_33_	GPa	53.19
G_23_	GPa	21.05
G_31_	GPa	21.05
G_12_	GPa	22.57
*v*_23_		0.4402
*v*_13_		0.4402
*v*_12_		0.3500
*ρ*	kg/m^3^	7750

**Table 3:** Stress, strain and stiffness of the silicone rubber at each load increment.

Static Load (N)	Stress (kPa)	Strain	Stiffness (kPa)
0.5	1.2875	0.01563	61.757
1.0	2.5375	0.03498	67.473
1.5	3.7875	0.05281	72.740
2.0	5.0375	0.06943	77.650
2.5	6.2875	0.08506	82.267

**Table 4:** Dynamic stiffness of the silicone rubber at each load increment.

Static Load (N)	Dynamic Stiffness (kPa)
0.5	431.27
1.0	472.25
1.5	509.12
2.0	543.46
2.5	575.78
